# 2190. Lack of Knowledge of Antibiotic Risks Among an Outpatient Population is Associated with Intention to Use Non-Prescription Antibiotics

**DOI:** 10.1093/ofid/ofad500.1812

**Published:** 2023-11-27

**Authors:** Eva Amenta, Barbara Trautner, Kiara Olmeda, Juanita Salinas, Monisha Arya, Lindsey Laytner, Michael Paasche-Orlow, Larissa Grigoryan

**Affiliations:** Baylor College of Medicine, Houston, Texas; Michael E. DeBakey Veterans Affairs Medical Center / Baylor College of Medicine, Houston, TX; Baylor College of Medicine, Houston, Texas; Baylor College of Medicine, Houston, Texas; Baylor College of Medicine, Houston, Texas; Baylor College of Medicine, Department of Family and Community Medicine, Houston, TX; Tufts University, Boston, Massachusetts; Baylor College of Medicine, Houston, Texas

## Abstract

**Background:**

Antibiotic use without a prescription (non-prescription use) leads to risks for patients and the public, including adverse drug reactions,

*Clostridoides difficile*

infections, microbiome disruption, and the development of antimicrobial resistance. We hypothesized that patients’ lack of knowledge of risks associated with antibiotic use would be associated with non-prescription use.

**Methods:**

We surveyed patients in public clinics and private emergency department waiting rooms in Houston from January 2020 through June 2021. Respondents’ knowledge about risks associated with antibiotic use was assessed with an open-ended question, “Do you know about any risks associated with antibiotic use?” Intended use was defined as endorsing an intention to use antibiotics without a prescription. The effects of age, gender, race/ethnicity, education, and knowledge of the risks associated with antibiotics on intended use were investigated using logistic regression.

**Results:**

We surveyed 564 individuals (median age of 51), with 72% identifying as female, 46.6% as Hispanic and 33% as Black or African American (Table 1). When asked about knowledge of antibiotic risks, 354 (63%) respondents were able to report at least one risk associated with antibiotics. Risks were categorized into themes that included side effects of antibiotics (270/564, 48%), antibiotic resistance (19%), disruption of the microbiome (5%), drug-drug interactions (4%), fear of taking medication (4%), and other (0.05%) (Table 2). Lack of knowledge of antibiotic risks OR 1.48 (95% CI 1.04-2.10) and younger age (age 18-64 years) OR 2.24 (1.29-3.89) were independently associated with intention to use non-prescription antibiotics (Table 3).

**Figure:**
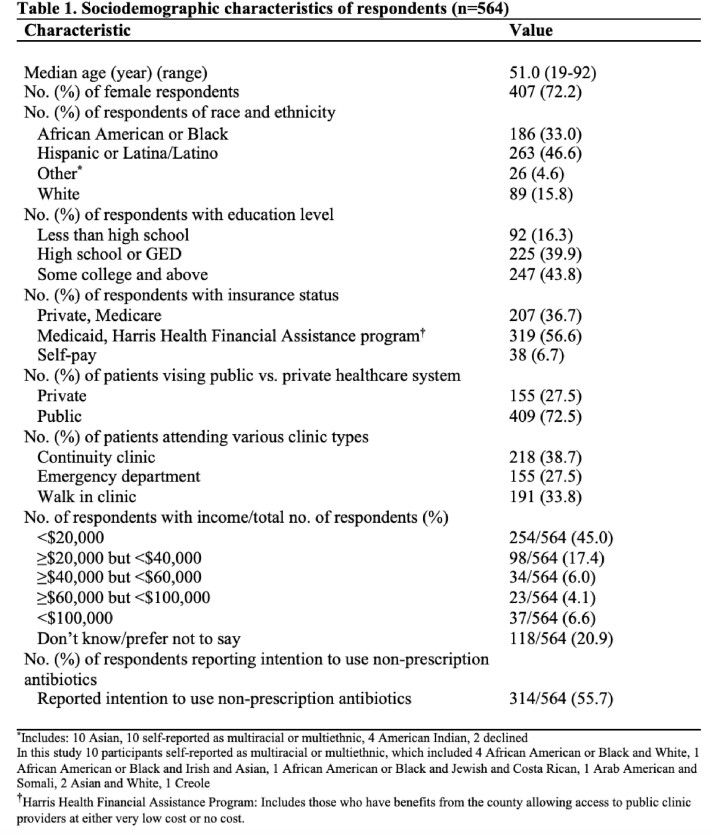

**Figure:**
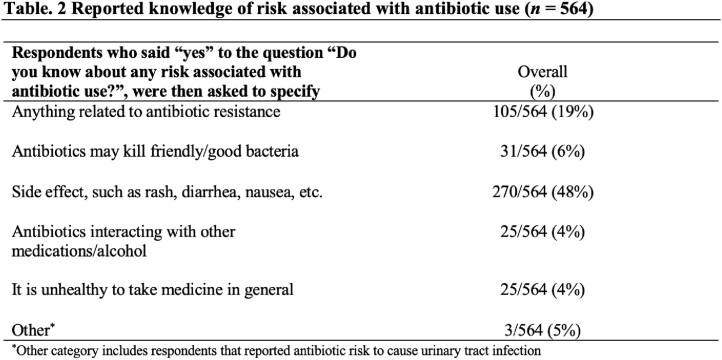

**Figure:**
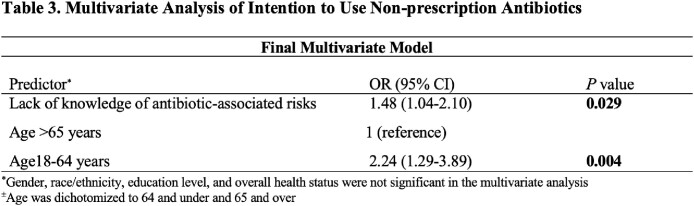

**Conclusion:**

Over one-third of respondents reported no knowledge of antibiotic-associated risks. Only 19% of respondents were aware of the concept of antimicrobial resistance. Lack of knowledge was associated with the intention to use non-prescription antibiotics, suggesting that further patient education about potential downsides of taking antibiotics is essential to reducing non-prescription antibiotic use.

**Disclosures:**

**Barbara Trautner, MD, PhD**, Genentech: Grant/Research Support|Peptilogics: Grant/Research Support

